# Draft genome sequence of biofilm-forming methicillin-resistant *Staphylococcus aureus* MTR_V1 strain isolated from a ready-to-eat food in Bangladesh

**DOI:** 10.1128/MRA.00597-23

**Published:** 2023-09-15

**Authors:** Fatimah Muhammad Ballah, Md. Saiful Islam, Samina Ievy, Farhana Binte Ferdous, Md. Abdus Sobur, AMM Taufiquer Rahman, Marzia Rahman, M. Nazmul Hoque, Jayedul Hassan, Md. Tanvir Rahman

**Affiliations:** 1 Department of Microbiology and Hygiene, Faculty of Veterinary Science, Bangladesh Agricultural University, Mymensingh, Bangladesh; 2 Naogaon District Hospital, Naogaon, Bangladesh; 3 Department of Gynaecology, Obstetrics, and Reproductive Health, Bangabandhu Sheikh Mujibur Rahman Agricultural University, Gazipur, Bangladesh; University of Maryland School of Medicine, Baltimore, Maryland, USA

**Keywords:** whole-genome sequencing, MRSA, biofilm formation, ready-to-eat food, CRISPR arrays, antibiotic resistance genes, virulence factor genes, Bangladesh

## Abstract

This announcement provides the genome sequence of the biofilm-forming methicillin-resistant *Staphylococcus aureus* MTR_V1 strain isolated from a ready-to-eat food sample in Bangladesh. Our assembled genome had a length of 2.8 Mb, 27 contigs, two CRISPR arrays, 38 predicted antibiotic resistance genes, and 66 predicted virulence factor genes.

## ANNOUNCEMENT

Excessive antibiotic use has caused antibiotic resistance, resulting in diverse multidrug-resistant (MDR) strains that pose a significant global health risk (1, 2). The development of resistance and biofilm in methicillin-resistant *Staphylococcus aureus* (MRSA) strains presents a severe threat to human health (3, 4).

Between June 2021 and March 2022, ready-to-eat food samples were collected from various vendors and restaurants in Mymensingh district, Bangladesh. The samples were processed using previous protocols (4) and incubated overnight in nutrient broth (HiMedia, India) at 37°C. The samples were then streaked on Mannitol Salt Agar (HiMedia, India) media, and the resulting colonies underwent staining and biochemical tests to isolate *Staphylococcus aureus* (5). *S. aureus* was identified through the matrix-assisted laser desorption ionization time-of-flight mass spectrometry assay (6). MRSA was confirmed by detecting the *mecA* gene, which was amplified by the primers *mec*A1 (5′-AAAATCGATGGTAAAGGTTGGC) and *mec*A2 (5′-AGTTCTGCAGTACCGGATTTGC) (7). Biofilm formation in MRSA was determined using Congo Red Agar (8) and Crystal Violet (9) assays. Finally, a biofilm-forming MRSA strain, *S. aureus* MTR_V1, was selected and incubated in nutrient broth (HiMedia, India) overnight at 37°C, and the harvested culture was used for DNA extraction with a Qiagen DNA mini kit (Qiagen, Hilden, Germany). The extracted DNA from the isolate was then subjected to enzymatic fragmentation using NEBNextdsDNA Fragmentase Kit (NEB, MA, USA) according to the manufacturer’s instructions. The fragmented DNA was subjected to size selection using SPRI beads, which helped in isolating DNA fragments of the desired size range for sequencing (10). The sequencing library was created using the Nextera DNA Flex library prep kit (Illumina, San Diego, CA, USA) and sequenced on the Illumina NextSeq2000 platform with paired-end reads (2  ×  150). The genome was assembled using the Unicycler.v0.4.9 (11), and the raw paired-end reads (*n* = 9,253,470) were trimmed using Trimmomatic.v0.39 (12). FastQC.v0.11.7 (13) was used to evaluate the quality of the trimmed reads. The genome was annotated using PGAP.v3.0 (14). SCCmecFinder.v1.2 (15) was employed for determining the predicted *SCCmec* element; PlasmidFinder.v2.1 (16) for plasmid typing; CARD.v3.2.4 (17), NDARO.v2023 (18), ResFinder.v4.1 (19), and PATRIC.v3.2.76 (20) for antibiotic resistance genes (ARGs); VFDB (21), VirulenceFinder.v2.0 (22), and Victors (23) for virulence factor genes (VFGs); MLST.v2.0 (24) for sequence type; DrugBank.v4.0 (25) and TTD (26) for drug target genes (DTGs); TCDB (27) for transporter genes (TGs); and RAST.v2.0 (28) for metabolic functional features. Default parameters were used for all software used in this study, unless otherwise specified.

The *S. aureus* MTR_V1 genome assembly contained 27 contigs with a G + C content of 32.65% and three contig L50s with an N50 value of 280,895 bp. It had a genome size of 2,766,668  bp and a coverage of 30.0×. Our genome also harbored 2,703 CDS, 64 RNA genes, and 89 pseudogenes. The *SCCmec* element of the *S. aureus* MTR_V1 strain was predicted as SCCmec_type_IVa(2B), and the MLST analysis assigned it as sequence type ST1930. Additionally, the assembled genome contained two CRISPR arrays, three plasmids, 38 predicted ARGs, 66 predicted VFGs, 35 DTGs, 97 TGs, and 273 subsystems (with 33% coverage and 1,231 genes).

## Data Availability

The Whole Genome Sequencing (WGS) shotgun analysis of *S. aureus* MTR_V1 was formally submitted to GenBank and can be accessed using the accession number JAPKJV000000000. The associated data, comprising the raw reads, have been deposited with the following accession numbers: BioProject—PRJNA902494, BioSample—SAMN31761769, and SRA—SRR25110733. In this article, the specific version indicated is JAPKJV000000000.1.

## AFTER EPUB

*[This article was published on 15 September 2023 with M. Nazmul Hoque's name misspelled as "Haque" in the byline. The byline was updated in the current version, posted on 19 October 2023.]*
